# Supplementary material for: Accessory genome of the multi-drug resistant ocular isolate of Pseudomonas aeruginosa PA34
Source: PLoS One. 2019 Apr 15;14(4):e0215038. doi: 10.1371/journal.pone.0215038 (PMC6464166; doi:10.1371/journal.pone.0215038)
Supplement: S1 Table — (DOCX) [file pone.0215038.s001.docx]

**Supporting information**

**S1 Table. Gaps in pseudogenome of PA34 with comparison with PA14.**

|  | **Pseudo-genome** | | **Reference genome (PA14)** | |
| --- | --- | --- | --- | --- |
| Gaps* | start on | end on | start on | end on |
| overlapping | 3254830 | 3254929 | 3157854 | 3089293 |
| overlapping | 3720994 | 3721093 | 3555358 | 3554586 |
| overlapping | 4717946 | 4718045 | 4551439 | 4425625 |
| overlapping | 6625799 | 6625898 | 6333379 | 6331788 |
| overlapping | 6810480 | 6810579 | 6516370 | END |

*The overlapping gaps are the joint between the contigs of draft genome. ABACAS comparison generates a gap of 100 NNN… indicating a junction between contigs and may not be a real gap.
