# Supplementary material for: Accessory genome of the multi-drug resistant ocular isolate of Pseudomonas aeruginosa PA34
Source: PLoS One. 2019 Apr 15;14(4):e0215038. doi: 10.1371/journal.pone.0215038 (PMC6464166; doi:10.1371/journal.pone.0215038)
Supplement: S2 Table — (DOCX) [file pone.0215038.s002.docx]

**Supporting information**

**S2 Table. Primer proposed for the gap validation.**

| Gap region starts | Forward primer | Tm  (˚C) | Reverse Primer | T_m_  (˚C) |
| --- | --- | --- | --- | --- |
| 3254830 | TGTAGCGGTGCATCAAGTAATG | 57 | TGTATCCGCTATGTTGAGGTCA | 56 |
| 3720994 | CTGCGGCAAAAGACTTTCAAAA | 56 | GACCAGGTCTATGCGTTCCT | 58 |
| 4717946 | GCATCCTATTGATCTTGCCTCG | 58 | GCGGCCTTGATTTTCAGAAAGA | 57 |
| 6625799 | CAGTCAGGGAAGCAGGTAAATTG | 57 | ACCTTGATGCGGTTGTTAACAC | 58 |
| 6810480 | CAAGCGTTTTGTGGGGATGG | 59 | TTCATCCTAAGCCTGTATCCGC | 58 |
